# Stenosis of the Inferior Vena Cava: A Murine Model of Deep Vein Thrombosis

**DOI:** 10.3791/56697

**Published:** 2017-12-22

**Authors:** Holly Payne, Alexander Brill

**Affiliations:** ^1^Institute of Cardiovascular Sciences, College of Medical and Dental Sciences, University of Birmingham

**Keywords:** Immunology, Issue 130, deep vein thrombosis, murine model, inferior vena cava, stenosis

## Abstract

Deep vein thrombosis (DVT) and its devastating complication, pulmonary embolism, are a severe health problem with high mortality. Mechanisms of thrombus formation in veins remain obscure. Lack of mobility (*e.g.*, after surgery or long-haul flights) is one of the main factors leading to DVT. The pathophysiological consequence of the lack of mobility is blood flow stagnation in venous valves. Here, a model is described that mimics such flow disturbance as a thrombosis-driving factor. In this model, partial flow restriction (stenosis) in the inferior vena cava (IVC) is created. Closure of about 90% of the IVC lumen for 48 h results in development of thrombi structurally similar to those in humans. The similarities are: i) most of the thrombus volume is red, i.e., consists of red blood cells and fibrin, ii) presence of a white part (lines of Zahn), iii) non-denuded endothelial monolayer, iv) elevated plasma D-Dimer levels, and v) possibility to prevent thrombosis by low molecular weight heparin. Limitations include variable size of thrombi and the fact that a certain percentage of wild-type mice (0 - 35%) may not produce a thrombus. In addition to visual observation and measurement, thrombi may be visualized by non-invasive technologies, such as ultrasonography, which allows for monitoring the dynamics of thrombus development. At shorter time points (1 - 6 h), intravital microscopy may be applied to directly observe events (*e.g.*, recruitment of cells to the vessel wall) preceding thrombus formation. Use of this method by several teams around the world has made it possible to uncover basic mechanisms of DVT initiation and identify potential targets that might be beneficial for its prevention.

**Figure:**
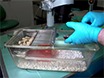


## Introduction

Deep vein thrombosis (DVT) is the development of thrombi in deep veins, usually (but not only) in legs. In conjunctions with pulmonary embolism (PE; designated together as Venous Thromboembolism, VTE) it develops in approximately 900,000 Americans annually and represents a serious health issue and economical problem^1^,^2^. PE, a complication of DVT, occurs when a thrombus gets detached from its initial location and reaches the lungs, which may cause respiratory insufficiency and death. The death toll from VTE exceeds mortality from AIDS, breast cancer and traffic accidents, combined^3^.

The major factor causing DVT besides known reasons, such as cancer or trauma, is the lack of mobility 4,5. This may result from surgery (especially orthopedic), paralysis, long-haul flights or other reasons. Blood flow in veins depends on the muscle pump and therefore limb immobilization results in stagnant blood flow in venous valves, which leads to thrombosis. The purpose of the method described herein is to recapitulate such blood flow distortion^6^,^7^. Partial flow restriction in the inferior vena cava (IVC) mimics conditions created in human venous valves and results in the formation of a thrombus similar in structure to human thrombi^6^. The large part of a thrombus is red and consists of red blood cells, fibrin and incorporation of platelets. Thrombi have a small "white part" enriched in platelets (Figure 1), which resemble "lines of Zahn" described in human venous thrombi. Both parts of the thrombus contain also neutrophils^8^, which are among the first cells to be recruited at the site of future thrombus^6^,^9^. Neutrophils in the red part expel neutrophil extracellular traps (NETs), whereas neutrophils in the white part seem to be devoid of NETs^8^. Similarly to human DVT, thrombosis in mice is accompanied by elevated plasma D-dimer levels (Figure 2). Low molecular weight heparin (enoxaparin), used for DVT prophylaxis in patients, also prevents thrombosis in mice. An important advantage of this method is absence of endothelial denudation^9^, which is characteristic of human DVT^10^. This feature makes IVC stenosis a more clinically relevant model of DVT than, for example, induction of thrombosis by ferric chloride, which induces endothelial denudation and in which thrombi consist predominantly of platelets^11^,^12^,^13^. A model of complete stasis in the IVC is preferred by several teams^14^,^15^,^16^. In contrast to stenosis, in which residual flow is maintained in the vessel, application of stasis completely stops the flow and thus limits the accessibility of systemically administered substances to the thrombosis site. Also, it seems that mechanisms underlying thrombosis induced by stenosis and stasis are different: stenosis results in the development of local inflammation (activation of the endothelium, release of Weibel-Palade body content, recruitment of immune cells and platelets to the vessel wall) causing "immunothrombosis"^6^,^9^,^17^, whereas stasis seems to induce rather thrombosis based, in particular, on tissue factor and other coagulation and fibrinolysis-related mechanisms^18^,^19^,^20^. Thus, the stenosis and stasis models reflect slightly different aspects of venous thrombus development, although their mechanisms may certainly overlap. Electrolytic IVC model (EIM) of DVT^21^,^22^ induces a thrombus only partially occluding the vessel wall. Therefore, it is convenient for testing the effects of systemic administration of different drugs on thrombus growth. This model, however, assumes disruption of the IVC wall integrity (insertion of a needle) and induction of thrombosis by electric current making the pathophysiological relevance of this mechanism at least disputable.

## Protocol

All animal procedures were approved by Animal Welfare Ethical Review Body and the UK Home Office (UK, Project License 40/3745). Mice on C57BL/6 background, 8 - 12 week old, 19 - 25 g body weight, of both genders are used.

### 1. Animal Anesthesia

1. Place a mouse into an induction chamber and induce anesthesia by a mixture of 5% isoflurane with 100% oxygen. Use a flow rate of 0.5 L/min. Wait until the mouse completely stops moving and its frequency of breathing becomes rare.
2. Remove the mouse from the chamber and shave the abdomen with an electric clipper. Remove hair from the abdomen as thoroughly as possible to avoid contamination of the abdominal cavity.
3. **Place the mouse in the supine position and place mouse's nose into a cone linked to the anesthesia system. Decrease the percent of isoflurane to 2-3% (the exact percent may slightly differ from animal to animal). Make sure the animal is sleeping with its respiratory rate being lower than usual but avoiding gasping. If gasping occurs, decrease the percent of isoflurane by 0.5%.**
  1. Check the pedal reflex to confirm proper anesthetization and start surgery only if it is negative. Apply vet ointment on the eyes to prevent dryness.
4. Apply an anti-bactericidal solution (1% Hibitane) on the mouse's abdomen and wipe the area using a sterile cotton bud in a way excluding potential re-contamination of the surgical site (*e.g.*, inside to out in a circular fashion). Repeat 3 times.

### 2. Application of the IVC Stenosis and Mouse Recovery

NOTE: All the instruments as well as materials (cotton buds, gauze, saline *etc.*) coming into contact with the surgery area must be sterile. The operator must scrub up prior to surgery and wear sterile gloves and gown during the procedure. Microscope handles should be wrapped in a sterile foil.

1. Give mice an anti-pain treatment prior to surgery and during the entire experimental period. For example, use buprenorphine 0.1 mg/kg subcutaneously 30 min before surgery and then every 12 hours until the animal is euthanized. As thrombosis in this method develops similarly to sterile inflammation, avoid using anti-inflammatory drugs as a premedication.
2. Cover the mouse with a sterile drape and make a hole in the drape to expose the site of surgery. Using scissors, make an incision along the abdominal midline, 1.5 cm down from the sternum. Using cotton buds exteriorize the intestines to the left-hand side of the mouse and cover them with sterile gauze soaked in warm (37 °C) saline to avoid drying out.
3. **Identify the IVC and its side branches (there may be 0, 1 or 2 of them) in the caudal direction from the site of fusion of the IVC with the left renal vein. Ligate all visible side branches with 7-0 inert Prolene sutures as close to the IVC as possible.**
  1. Try not to hold the side branches with the forceps directly but rather pull them up holding the fat tissue surrounding them with forceps in one hand and make a tunnel beneath the branch with forceps in the other hand. Do not close back branches.
4. Holding surrounding tissues with forceps, pull the IVC up and left producing tension in the small area between the IVC and the aorta **exactly** at the angle between the IVC and the left renal vein. Take into account that it is virtually impossible to separate the aorta from the IVC even 2-3 mm below (in the caudal direction).
5. Using diverging movements with forceps tips in your right hand make a hole between the aorta and the IVC. Keep in mind that the more movements made, the higher the chance of damaging the vessel. Ideally, try to minimize the number of movements to 3-5.
6. Prepare a piece of 7-0 inert Prolene suture of about 2 cm long. Place it in the mouse's peritoneal cavity on the operator's left-hand side in the vicinity to the IVC.
7. Pull the IVC up and left again. Insert your right forceps tips into the hole between the aorta and the IVC so that the tips appear on the other side of the IVC. Take the suture and pull it back through the hole (Figure 3**A**).
8. Make a provisional knot with the suture but do not close it. Place 30G needle ("a spacer") bent as a hook into the knot and now close it (Figure 3**B**).
9. Remove the spacer (Figure 3**C**).
10. Return the intestines back to the peritoneal cavity with a cotton bud and distribute them equally in it.
11. Close peritoneum with 6-0 vicryl suture using continuous loops.Close the first and last loops as a knot.
12. Close skin using metal staples.
13. Inject the mouse with 0.5 mL of warm (37 °C) glucose saline subcutaneously to restore the liquid balance of the organism.
14. Place the mouse into an individual cage in a warm (25 °C) chamber or room and put food and gel water inside the cage. Observe until the mouse is fully recovered (generally, around 1 h).

## Representative Results

Herein, a model of DVT induced by flow distortion in the IVC is described. Induction of stenosis in the IVC initiates distortion and stagnation of the blood flow and after a while (in this case, 48 h) results in the development of a thrombus, structurally similar to human DVT thrombi (Figure 1**A**). The presence of a thrombus inside the IVC is easily detectable visually (Figure 1**B**). An important similarity between the model and human DVT is elevated plasma D-dimer levels (Figure 2). D-dimer is a product of fibrin degradation and its presence in the blood suggests that an active thrombotic process is going on.

The model is presented step-by-step in Figure 3**A-C**. The IVC is carefully exposed, a hole between the IVC and the aorta is made and a suture (Prolene 7-0) is pulled through the hole beneath the IVC. Then the IVC is ligated by the suture over a spacer (30G needle, Figure 3**D**), after which the spacer is removed. This procedure allows for about 90% closure of the IVC lumen leaving the remaining 10% patent. This ensures blood flow stagnation eventually leading to thrombosis.

Figure 4 demonstrates the major disadvantage of the model, variable thrombus size. All these thrombi were obtained in the same experiment performed on wild type male mice of the same origin, age and similar body weight. Although all of the mice produced thrombi (thrombosis prevalence of 100%), their size clearly varies in a wide range.

**Figure:**
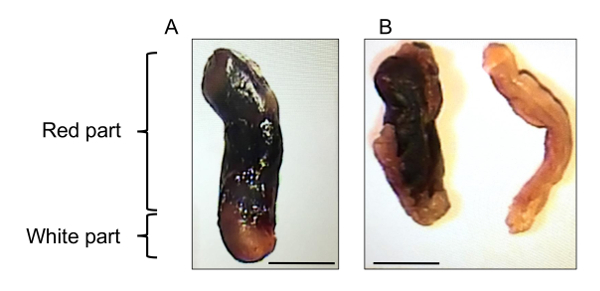

Figure 1:** Representative thrombus after 48 h restriction/stenosis. **(**A**) a typical thrombus after 48 h IVC stenosis. Note red (red blood cell-enriched) and white (platelet-enriched) parts. (**B**) IVC with (left) or without (right) a thrombus. Scale bars = 2 mm. Please click here to view a larger version of this figure.

**Figure:**
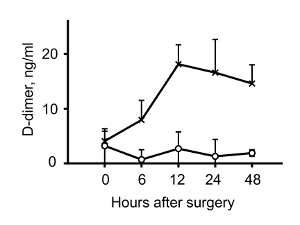

Figure 2**: Elevated plasma D-dimer levels after IVC stenosis application. **Mice were subjected to IVC stenosis. Blood was taken at the indicated time points, stabilized 1:9 with 3.8% sodium citrate, plasma was prepared by centrifugation (2,300 x g, 5 min) and D-dimer levels were determined by an ELISA kit according to manufacturer's instructions. Circles designate sham-operated mice, crosses designate IVC stenosis. Please click here to view a larger version of this figure.

**Figure:**
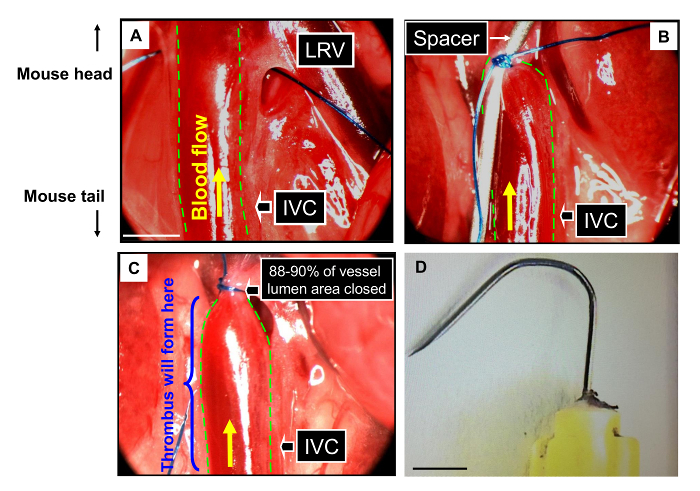

Figure 3:** Flow restriction/stenosis in the Inferior Vena Cava (IVC) model of DVT in mice. **Consecutive stages of the model are presented. (**A**) a hole between the IVC and the aorta is made and a suture is pulled through it around the IVC. (**B**) a knot is closed over a spacer (30 G needle). (**C)** the spacer is removed: the final view that the surgeon sees before closing the mouse. The dashed yellow line demarcates the IVC. LRV stands for left renal vein. Scale bars = 0.2 mm. (D) the spacer (30 G needle). Scale bars =  2 mm. Please click here to view a larger version of this figure.

**Figure:**
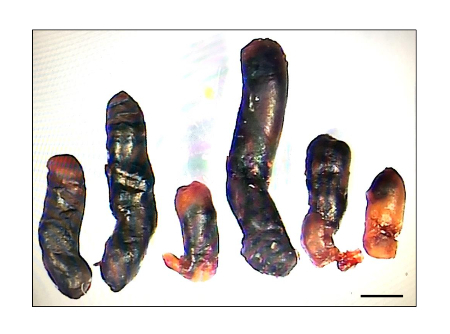

Figure 4**. Variability of thrombus size. ** Mice were subjected to 48 h IVC stenosis. Presented are thrombi obtained in the same experiment. Scale bars 2 mm. Please click here to view a larger version of this figure.

## Discussion

Here, a protocol of the IVC stenosis, which mimics blood flow distortion, a major triggering factor for DVT, is presented. Stenosis of the IVC induces development of thrombi in 65-100% of C57BL/6 mice within 48 h and 25-50% of mice within 2-6 h^6^,^8^,^23^ (Brill A, unpublished data, 2016). A major limitation of the method is the variability in thrombus size^24^ (Figure 4), which is observed after both short-term (6h) and long-term (48 h) IVC stenosis. The reasons for such variability (given that the same conditions, such as the spacer, anesthesia etc., are used) remain obscure but one may speculate that anatomical variations between the mice (e.g., width of the IVC, number and location of both side and back branches) underlie this phenomenon. The variability in thrombus size makes thrombosis prevalence (percent of mice with a thrombus) the main outcome. Thrombosis prevalence can be compared using a contingency table and the Fisher's exact test. One of the experiments was an exception when reduced thrombus size with the same thrombosis prevalence after injection of podoplanin-inhibiting antibodies was observed 7.

This method is especially useful when venous thrombosis initiation is studied. It allows for investigating early events in the vessel wall, such as recruitment of cells, leading eventually to thrombosis. Pro- or anti-thrombotic effects of drugs can be evaluated using this model with thrombosis prevalence being a primary readout. Stenosis for 48 h is applicable to reveal an anti-thrombotic phenotype, whereas 6 h stenosis can be used if a prothrombotic phenotype is expected. Histological analysis of the thrombus and surrounding IVC wall can also be performed.

The question whether side branches should be ligated or left patent remains open. One group has shown that ligation of side branches does not increase thrombus size and location of a side branch closer than 1.5 mm to the IVC ligation site dramatically impairs thrombus development^25^. Closure of side branches may induce endothelial injury in them and also increase the time of surgery^26^. In our hands, lack of side branch closure substantially decreases thrombosis prevalence (down to 10 - 30% after 48 h stenosis; Brill, unpublished) and therefore we ligate all visible side branches.

Ideally, littermate controls should be used as mice, even on the same background but from different sources, may have slightly different thrombosis prevalence. If the effect of DVT itself on any parameters (for example, biochemical) is studied, sham-operated animals should be used. Sham-operated mice undergo the same procedure but the ligature around the IVC is closed loosely and left there without producing stenosis.

The most frequent mistake (a critical step) in this protocol is an effort to separate the aorta and the IVC not precisely at the angle between the vessels but slightly lower, which usually results in bleeding. When a massive bleeding occurs, good recovery of the mouse becomes unlikely and it is recommended to stop the experiment and euthanize the animal. Normally, mice recover well, move inside the cage and do not substantially lose weight. We recommend using mice above 20 g for both males and females and keep animals (especially males) in individual cages after surgery until the end of the experiment to avoid fighting and injury. It has been reported in another (electrolytic) model of DVT that male mice produce larger thrombi than females^27^. Analysis of our data has not revealed substantial difference in thrombosis prevalence between male and female mice (Brill, unpublished). Therefore, researchers are encouraged to perform experiments using the IVC stenosis model on mice of both genders.

Unravelling of sutures and/or staples may not be ruled out especially in long experiments (a week and longer). Thus, mice should be checked at least twice a day specifically for suture integrity and a special attention should be given to the appearance of blood traces on the cage bedding.

It should be noted that, like any other animal model, stenosis of the IVC has its limitations in terms of translation into humans. For example, murine IVCs do not have valves, whereas human DVT develops inside venous valves. Also, humans have vertical spinal orientation with the muscle pump being an important mechanism accelerating blood flow in veins. In contrast, mice have horizontal spinal orientation with no role of the muscle pump in supporting blood return to the heart. These limitations should be considered when translating mouse data to the human disease.

## Disclosures

The authors have nothing to disclose.
